# Intraoperative Elastography in Pancreatic Cancer—Clinical Applications and Systematic Review of the Literature

**DOI:** 10.3390/cancers18030473

**Published:** 2026-01-31

**Authors:** Miana Gabriela Pop, Cristina Pojoga, Ioana Bartoș, Florina Gabor-Harosa, Sandu Brînzilă, Caius Mihai Breazu, Adrian Bartoș

**Affiliations:** 1 Department of Public Health and Management, Faculty of Medicine, Iuliu Hațieganu University of Medicine and Pharmacy Cluj-Napoca, Victor Babeș Str., No. 8, 400012 Cluj-Napoca, Romania; miana.hosu@umfcluj.ro (M.G.P.); florina.gabor@umfcluj.ro (F.G.-H.); 2 Regional Institute of Gastroenterology and Hepatology, Croitorilor Str., No. 19-21, 400162 Cluj-Napoca, Romania; 3 Department of Clinical Psychology and Psychotherapy, Babeș-Bolyai University, Sindicatelor Str., No. 7, 400029 Cluj-Napoca, Romania; 4 Regina Maria Health Network, Dorobanților Str., No. 29, 400609 Cluj-Napoca, Romania; iancuioana2011@gmail.com (I.B.); sandubrinzila91@gmail.com (S.B.); adrian.bartos@ubbcluj.ro (A.B.); 5 Regional Institute of Urology and Renal Transplant Cluj-Napoca, Clinicilor Str., No. 4-6, 400006 Cluj-Napoca, Romania; 6 Department of Anesthesia and Intensive Care, Cluj County Emergency Clinical Hospital, Clinicilor Str., No. 4-6, 400349 Cluj-Napoca, Romania; caius.breazu@umfcluj.ro; 7 1st Department of Anesthesia and Intensive Care, Faculty of Medicine, Iuliu Hațieganu University of Medicine and Pharmacy Cluj-Napoca, Victor Babeș Str., No. 8, 400012 Cluj-Napoca, Romania; 8 Institute for Advanced Studies in Science and Technology—STAR-UBB Institute, Babeș-Bolyai University Cluj-Napoca, Mihail Kogalniceanu Str., No. 1, 400084 Cluj-Napoca, Romania

**Keywords:** intraoperative elastography, pancreatic tumor, postoperative pancreatic fistula, pancreatic surgery

## Abstract

**Background**: Pancreatic cancer (PC) is expected to be the second leading cause of cancer-related death by 2030. Surgical resection with R0 margins remains the only available treatment capable of improving the overall survival of the patients; thus, appropriate characterization of pancreatic tumors is mandatory for the correct assessment of PC resectability. Despite advances in pancreatic surgery, POPF remains a frequent and dreaded complication that impacts the morbidity and mortality of PC patients, entailing both clinical and economic consequences. Soft pancreatic texture is known as an independent risk factor for POPF occurrence in pancreatic surgery. Intraoperative exploration of the pancreas is most frequently assessed subjectively, through the surgeon’s palpation. Intraoperative elastography is a modern ultrasound technique suitable to replace the surgeon’s intraoperative palpation to better evaluate pancreatic lesions, pancreatic texture, and improve surgical management. Thus, intraoperative elastography could provide quantifiable and reproducible information in pancreatic parenchyma characterization. Real-time intraoperative assessment of pancreatic texture through an objective method could improve surgical decisions. This systematic review analyzes the role of intraoperative elastography in differentiating benign from malignant pancreatic tumors and the efficacy of this technique in the assessment of pancreatic texture as a predictor of postoperative pancreatic fistula (POPF). **Methods**: We conducted a comprehensive systematic literature research on PubMed, Google Scholar, Scopus, Web of Science, Embase and Cochrane Library Database using PRISMA framework guided by the words “intraoperative elastography” or “intraoperative elasticity imaging” or “intraoperative shear wave elastography” or “intraoperative strain elastography” and “pancreatic cancer” or “pancreatic neoplasm” or “pancreatic adenocarcinoma” or “pancreatic tumor” or “pancreatic fistula” or “postoperative pancreatic fistula” or “pancreatic leak”. Articles that were listed between 2000 and 2025 and written in the English language were screened for potentially relevant articles. The primary outcome was to evaluate the use of intraoperative elastography in differentiating between benign and malignant lesions of the pancreas. The second outcome was to assess the role of intraoperative elastography in the evaluation of pancreatic texture as a predictive factor for the occurrence of postoperative pancreatic fistula. **Results**: From a total of 17 publications, 2 scientific articles were considered relevant for the role of intraoperative elastography in differentiating benign from malignant pancreatic lesions, while 4 articles analyzed the role of intraoperative pancreatic elastography as a predictor of postoperative pancreatic fistula (POPF). Based on the results, detection of pancreatic cancer through intraoperative SWE is possible at cut-off values of 3 m/s and 28.7 kPa, and values of 2.2 m/s or less obtained after intraoperative elastography of the pancreas are considered an independent risk factor for POPF in pancreatic surgery. Reported cut-off values should, however, be interpreted as exploratory and should represent a starting point for further studies aimed at validating their clinical implementation. **Conclusions**: Intraoperative elastography can be a promising tool in pancreatic tumor characterization and could differentiate between benign and malignant pancreatic tumors and predict the risk of POPF, but further prospective studies are required before cut-off values can be routinely applied in clinical practice.

## Simple Summary

Pancreatic cancer (PC) is an aggressive disease that is expected to be the second leading cause of cancer-related death by 2030. Overall survival of patients with PC is often limited to only a few months, since most of the patients present with locally advanced tumors. Radical surgical resection with R0 margins remains the only available curative treatment, capable of improving survival rates in patients with PC; thus, appropriate characterization of pancreatic tumors is mandatory for correct assessment of PC resectability. Moreover, surgical management in pancreatic surgery must take into consideration the texture of the pancreas. Postoperative pancreatic fistula (POPF) is a major complication that can negatively influence morbidity and mortality in pancreatic surgery, and soft pancreatic texture is considered an independent risk factor for POPF. Unfortunately, evaluation of pancreatic parenchyma is mainly performed via subjective methods (like intraoperative surgeon’s palpation). Intraoperative elastography has been used in PC lesions characterization and real-time evaluation of the pancreatic parenchyma, and could be a promising tool in assessing pancreatic stiffness and predicting the risk of POPF in pancreatic surgery.

## 1. Introduction

### 1.1. Intraoperative Elastography

Intraoperative elastography is a modern ultrasound technique that tests the elasticity of the examined tissue [1]. Due to a lack of inter-reference factors, better results are obtained with an intraoperative approach, compared with transabdominal examination [1]. The use of elastography for pancreatic evaluation is sustained by the Ultrasound Guidelines of the Japanese Society of Medical Ultrasonics and the recent British Medical Ultrasound Guidelines for Professional Ultrasound Practice of Elastography in Non-hepatic Evaluation [2,3]. Two methods of elastography have been described: strain elastography (SE) and shear-wave elastography (SWE) [2,4]. Strain elastography (SE) is a quasi-static elastography method based on tissue deformation after compression of the region of interest; the result encompasses a colored map that is inversely related to tissue stiffness and can only be interpreted in a subjective manner [3]. As a prerequisite for performing SE, the examined organ should be in line with the probe and the aorta [4]. In shear-wave elastography (SWE), a transverse wave is generated by the acoustic radiation force impulse (ARFI), and, subsequently, tissue stiffness is measured according to the propagation speed of the wave [4]. Aortic pulsation can interfere with ARFI, especially at the level of the pancreatic body [2,3]. Intraoperatively, elastography has been used for liver tumors [5], where the method was found to be able to properly differentiate benign from malignant lesions and also for characterizing the degree of liver fibrosis [6,7]. Pancreatic stiffness using ARFI was evaluated transabdominally for normal and pathological pancreas; values of 1.3 m/s for normal parenchyma and greater values (up to 3.62 m/s) for diseases of the pancreas (acute and chronic pancreatitis, pancreatic tumors) were reported [8,9]. According to the Ultrasound Guidelines of The Japanese Society of Medical Ultrasonics, endoscopic ultrasound (EUS) pancreatic elastography is performed for malignant–benign distinction and evaluation of pancreatic fibrosis [2,10]. Results are supported by two meta-analysis of 1042 patients, reporting sensitivity rates of 95% (95% CI 93–96%), respectively, specificity rates of 69% (95% CI 63–75%) [11], and another of 1044 cases reporting similar sensitivity rates, 95% (95% CI 94–97%), and specificity rates, 67% (95% CI 61–73%) [12]. Limited data exists regarding the use of intraoperative pancreatic elastography and its role in pancreatic surgery.

### 1.2. Pancreatic Cancer

Pancreatic cancer (PC) is expected to be the second leading cause of cancer-related death by 2030 [13,14,15]. Known for its aggressive biology, PC is most frequently diagnosed as late-stage disease (either with distant metastases or locally advanced tumors), with limited overall survival of only a few months [16]. Promising results in recent studies have validated non-invasive methods that use biomarkers like serum exosome detection, has-let-7f-5p, for early diagnosis of metastatic PC [17]. Radical surgical resection with R0 margins remains the only available curative treatment, capable of improving survival rates in patients with PC [14,18]. Unfortunately, this is seldom achieved due to various foreseeable scenarios: distant metastases presented at the time of diagnosis, a percentage between 40 and 60% of non-metastasized cases present with borderline-resectable or locally advanced tumors [19,20], and high rates (up to 70%) of positive resection margins (R1) after surgical treatment with curative intent [21,22]. For borderline- and locally advanced PC, neoadjuvant chemotherapy with mFOLFIRINOX, gemcitabin and nab-paclitaxel regimens leads to increasing resectability rates [13,16,23]. After completion of systemic treatment, preoperative imaging evaluation is crucial to correctly identify patients who are candidates for surgery, given that approximately 30% of patients fail to proceed with surgery after neoadjuvant chemotherapy [24]. Subsequent neoadjuvant systemic therapy, pathologic changes like inflammation and fibrotic lesions emerge around the pancreatic tumor, aspects difficult to distinguish from the tumoral tissue, and, thus, overestimation of tumor extension and vascular invasion (including overestimation of superior mesenteric artery invasion) was documented [25,26]. The “halo” presented on preoperative imaging around the pancreatic tumor was found to allow for periarterial dissection of the superior mesenteric artery, an aspect documented also by Habib et al. [27]. In addition, preoperative CT evaluation sometimes fails in correctly evaluating the extension of PC, especially regarding the assessment of vascular invasion [28]. In conclusion, appropriate intraoperative examination is mandatory to better characterize PC and prevent omission of patients eligible for surgery.

### 1.3. Intraoperative Ultrasound in Pancreatic Cancer Surgery

Intraoperative ultrasound (IOUS) in PC surgery is gaining more significance due to its benefits: improved tumor localization and characterization, detection of small, undiagnosed PC lesions, assessment of tumor relationship with surrounding structures like the main pancreatic duct or great vessels, and assessment of surgical margins [21,29,30]. IOUS in PC surgery was found to influence surgical strategy in a percentage reaching up to 49–61% of the cases [31,32] and was found to be responsible for downstaging the resectability status of pancreatic tumors in up to one-third of the patients (ULTRAPANC study) [25,28]. Intraoperative ultrasound is considered a promising diagnostic tool because it can provide real-time high-resolution images while the transducer is placed directly on the organ of interest, the patient does not require any preparation, and the method is non-ionizing [31]. Due to its higher resolution images and direct approach, IOUS of the pancreas could better differentiate peritumoral fibrosis from tumoral tissue when compared with preoperative CT imaging [25]. Another advantage after IOUS evaluation of the pancreas is the reduction in intraoperative waiting times until frozen section results are provided [25]. All in all, IOUS can improve the staging of PC, improve the chance of obtaining R0 margins, offer detailed information regarding the peritumoral pathologic changes, like peritumoral fibrosis, and provide more accurate information about the presence of vascular invasion [33].

### 1.4. Postoperative Pancreatic Fistula (POPF)

Postoperative pancreatic fistula (POPF) is a frequent complication in pancreatic surgery that negatively influences morbidity and mortality of PC patients [34,35]. POPF is encountered in a percentage ranging between 13% and 50% of patients who undergo pancreatic surgery [35]. According to the International Study Group of Pancreatic Surgery (ISGPS), a rate of 14% of Grades B and C POPF (99% CI: 12–17%), and 23% Grades B and C POPF (99% CI: 17–30%) are presented after pancreatoduodenectomy, respectively, after distal pancreatectomies, with the highest rates presented after central pancreactectomies (20–60%) [36]. The presence of a soft pancreatic tissue is considered a critical independent risk factor for POPF in pancreatic surgery [34]. Other risk factors for POPF in pancreatic surgery are male gender, underlying pancreatic pathology, main pancreatic duct diameter, and blood loss [35]. Evaluation of pancreatic stiffness is currently performed via intraoperative palpation by the surgeon [34,35]. Pancreatic firmness is inversely correlated with POPF occurrence [35]. Intraoperative evaluation of the pancreas through the surgeon’s palpation is a subjective method, lacking objective elements, an aspect that limits the ability to reach valid scientific conclusions. Thus, intraoperative elastography of the pancreas could be a valuable tool to objectively assess pancreatic texture and its stiffness in order to better evaluate the risk of POPF. Intraoperative evaluation of the risk of POPF may determine changes in surgical strategy.

This paper aims to evaluate existing studies in the literature when intraoperative elastography of the pancreas was performed in pancreatic surgery, for either pancreatic tumor characterization or pancreatic texture assessment as a predictor of postoperative pancreatic fistula (POPF).

## 2. Materials and Methods

### 2.1. Study Design—Inclusion and Exclusion Criteria

This review was performed in accordance to the PRISMA (Preferred Reporting Items for Systematic Reviews and Meta-Analyses) guidelines and has not been registered.

Inclusion criteria: (1) studies evaluating the accuracy of intraoperative elastography in the differential diagnosis of benign and malignant pancreatic lesions, and (2) studies evaluating the ability of intraoperative elastography in predicting POPF after pancreatic resections.

Exclusion criteria: (1) incomplete data; (2) case reports.

### 2.2. Literature Search Strategy

We conducted a comprehensive systematic literature research on PubMed, Google Scholar, Scopus, Web of Science, Embase, and Cochrane Library Database using PRISMA guided by the words “intraoperative elastography” or “intraoperative elasticity imaging” or “intraoperative shear wave elastography” or “intraoperative strain elastography” and “pancreatic cancer” or “pancreatic neoplasm” or “pancreatic adenocarcinoma” or “pancreatic fistula” or “postoperative pancreatic fistula” or “pancreatic leak”. Articles that were listed between 2000 and 2025 were checked for eligibility. The primary outcome was to evaluate the use of intraoperative elastography in differentiating between benign and malignant lesions of the pancreas. The second outcome was to assess the role of intraoperative elastography in the evaluation of pancreatic texture as a predictive factor for the occurrence of postoperative pancreatic fistula.

### 2.3. Study Selection and Quality Assessment

Two reviewers (M.G.P and I.B) independently screened the titles and abstracts of the identified studies. Full text of potentially eligible studies was further screened independently and blindly by two reviewers (M.G.P and I.B). Both reviewers were responsible for data extraction (adult population undergoing pancreatic surgery, intervention: intraoperative elastography; outcome: tumor characterization and POPF occurrence; design of the study: retrospective or prospective) and further analysis. Any disagreements were further discussed, leading to a shared consensus with a third reviewer (A.B.).

The following data were extracted independently by two reviewers: study characteristics—first author, year of publication, study design (prospective or retrospective), and sample size (number of patients included in the study); elastography methods used (strain or shear-wave elastography), measurement units (m/s, kPa), and cut-off values; and outcomes—intraoperative pancreatic tumor characterization by elastography and POPF occurrence. In case of missing information, graphics were reviewed. Results were centralized in databases.

Outcomes for which data were sought: pancreatic stiffness assessed by intraoperative elastography (m/s or kPa) for pancreatic tumor characterization and normal pancreatic parenchyma; the incidence of postoperative pancreatic fistula (%) (POPF), defined according to the International Study Group of Pancreatic Surgery (ISGPS) criteria; and clinically relevant postoperative pancreatic fistula (Grade B or C). Elastography measurements: mean/median pancreatic stiffness (strain ratio, shear-wave velocity), cut-off values used to predict POPF or malignant pancreatic tumors.

Risk of bias and applicability concerns were assessed using the QADAS-2 tool. Two reviewers (M.G.P and I.B) independently evaluated four domains (patient selection, index test, reference standard, flow and timing), and disagreements were solved by a third reviewer (F.G.H).

Studies that assessed the use of intraoperative elastography in POPF prediction and pancreatic tumor characterization were tabulated separately, and key characteristics were recorded (design of the study, size of the sample, elastography modalities, cut-off values reported, sensitivity, specificity). Studies where data were missing were also reported in the corresponding table for clarity. Studies that reported different elastography measurements (kPa or m/s) were analyzed narratively. Studies reporting continuous measures were assessed using narrative synthesis.

Results were synthesized using a narrative approach (sensitivity and specificity at cut-off values were reported in only one study from each table, and, thus, the HSROC model could not be performed).

We anticipated study heterogenicity due to different elastography methods (strain and shear-wave elastography), reporting units (cut-off values reported as m/s and median values reported in kPa), design of the study (retrospective and prospective), and, even if subgroup analysis was taken into consideration, a limited number of studies did not allow for this type of analysis. Individual study results were examined to assess the difference between studies and taken into consideration when results were reported, allowing us to highlight sources of heterogeneity.

Due to the abovementioned heterogenicity and the limited number of studies, sensitivity analyses could not be realized.

Reporting bias was evaluated narratively by examining each study for selective outcome reporting.

The grade approach was used to assess confidence in 4 domains: risk of bias using QADAS-2, inconsistency, imprecision and potential bias.

## 3. Results

### Literature Search and Selection

**Figure 1:**
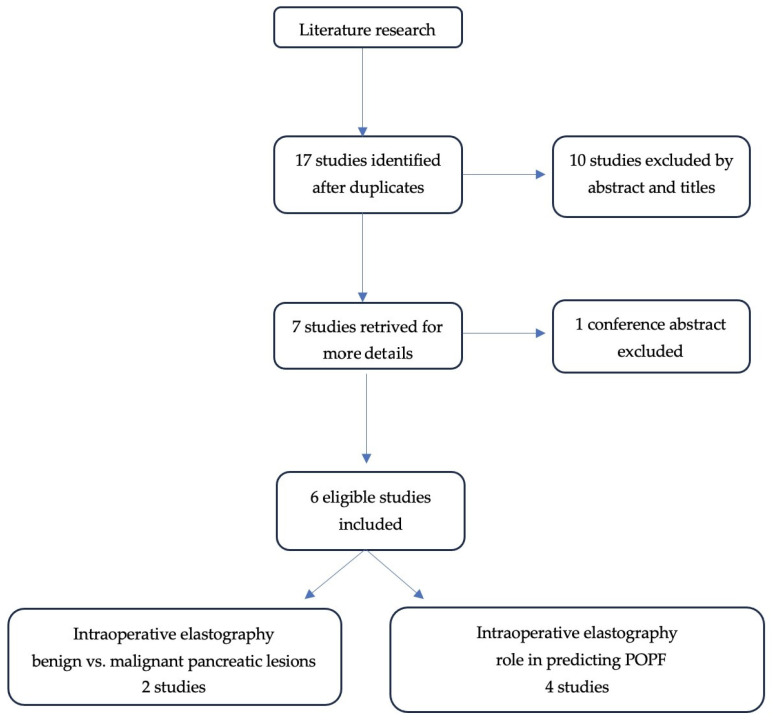
PRISMA diagram of the identified studies.

A total of 17 studies were initially identified by using the search strategy. Ten studies were excluded after screening of titles and abstracts. Of the seven studies left, six articles met the inclusion and exclusion criteria. The study selection process is shown in Figure 1.

Six scientific articles were included in the study, which were considered relevant for the role of intraoperative elastography in pancreatic cancer detection and postoperative pancreatic fistula prediction.

QADAS-2 assessment was independently performed by M.G.P and I.B. Disagreements were resolved by a third reviewer (F.G.H). Overall risk of bias across studies is presented in Table 1. Most studies show low risk of bias in patient selection and reference standard domains. The index test domain raised concerns mainly due to unclear blinding and non-predefined cut-off values.

The study characteristics of the included studies are shown in Table 2 and Table 3.

The use of intraoperative elastography for pancreatic stiffness evaluation with subsequent assessment of its role as a predicting factor in POPF occurrence was analyzed in four studies. Patients were included in a prospective manner in two of the studies [39,40], while for the other two, a retrospective analysis was performed [1,41].

The largest study evaluating the use of intraoperative elastography in pancreatic stiffness characterization and its role as a predictor of POPF was the one conducted by Kawamaba et al. A total of 48 patients who underwent pancreatoduodenectomy for pancreatic neoplasms were included in the study. Pancreatic adenocarcinoma was presented in 17 cases (35%), intraductal papillary mucinous neoplasm in 9 cases (19%), ampullary cancer in 6 patients (n = 13%), and distal bile duct cancer in 6 patients (13%). The mean age of included patients was 69 years (range, 26–91 years). A total of 34 males (71%) and 14 females (29%) were included. Intraoperative evaluation of pancreatic texture revealed 29 cases (60.4%) of soft pancreatic texture and 19 cases (40%) of hard pancreatic parenchyma. The main pancreatic duct diameter was 3 mm (0.5–9.9 mm). POPF was defined according to the International Study Group of Pancreatic Fistula (Grade B/C pancreatic fistula was considered POPF). POPF was recorded in 20 patients (41.6%). Mean elasticity of the pancreas was found to be 2.2 m/s (1.3–7.28 m/s). After multivariate analysis, pancreatic texture elasticity of 2.2 m/s or less was found to independently predict the risk of POPF after pancreatoduodenectomy performed for pancreatic neoplasm (*p* = 0.003).

Hatano et al. included in their study 41 patients; pancreatic stiffness and the risk of POPF were evaluated through intraoperative ultrasound elastography in pancreatic surgery: pancreatoduodenectomy (PD) was performed in 30 patients, while distal pancreatectomy (DP) was performed in 11 patients [40]. Patients included in the study were diagnosed with pancreatic neoplasms [40]. A total of 29 men and 12 women were included, with a median age of 71 years (43–82 years). Intraoperative ultrasound elastography was performed by the same examiner in three regions: at the site of pancreatic dissection, at the pancreatic head, and at the pancreatic tail sites. The mean values after three measurements at each site were recorded [40]. The median elastic ratio at the pancreatic resection site in the PD group was 1.82 m/s (1.53–2.25 m/s), *p* = 0.03, while in the DP group, the median elastic ratio at the pancreatic resection site was 1.98 m/s (1.62–2.18 m/s), *p* = 0.09 [40]. POPFs were diagnosed according to the International Study Group of Pancreatic Fistula. An amount of 11 POPFs appeared in the PD group (36.7%), while 7 POPFs were observed in the DP group (63.6%) [40]. After univariate analysis, an elastic ratio of 2.09 m/s or less was found to be a significant risk factor for POPF in the pancreatoduodenectomy group (*p* = 0.02) (but not in the distal pancreatectomy group) [40]. However, the results did not reach statistical significance in multivariate analysis [40].

Wada et al. evaluated pancreatic stiffness using both pre- and intraoperative elastography and analyzed its role as a predictor for POPF [1]. A total of 15 patients who underwent pancreatoduodenectomy for pancreatic neoplasms were included in the study. SWE was performed preoperatively (on the body of the pancreas) and intraoperatively in the same region; intraoperative SWE was compared with preoperative results. Quantitative parameters were recorded as the shear-wave elasticity index (kPa). Three POPFs (two Grade A and one Grade B) were identified after surgery. Even if lower SWE (corresponding to a softer pancreatic texture) was observed in patients who developed POPF, results did not reach statistical significance (*p* > 0.05) [1]. According to the results, higher SWE values correlated with higher levels of pancreatic fibrosis (Kruskal–Wallis test, *p* = 0.036) and with lower levels of daily output of pancreatic juice (Spearman’s rank correlation test, *p* < 0.028) [1].

In the study conducted by Yang et al., intraoperative elastography was used to characterize pancreatic texture. Ten patients with periampullary tumors were included in the study, who were further analyzed and compared with a control group (n = 9) [41]. Young’s module of the pancreatic neck was assessed with intraoperative ultrasound to determine pancreatic SWE. Results were correlated with preoperative plain CT scan values of the pancreas. The median value of SWE was 13.4 kPa (11.5 kPa, 17.7 kPa), with higher values in the periampullary tumors group, 16.0 kPa (11.4 kPa, 19.2 kPa), when compared with the control group, 13.2 kPa (11.5 kPa, 15.6 kPa); results did not reach statistical significance (*p* > 0.05) [41]. In addition, intraoperative SWE of the pancreas was found to negatively correlate with preoperative plain CT scan value, which was 34 ± 12 (*p* = 0.014) [41].

Certainty of evidence for the use of pancreatic elastography in predicting POPF is low to moderate. Even if the four studies assess the primary outcome, overall confidence is limited by small sample sizes and heterogeneity of the data. Direct comparison of the results between studies should not be attempted, and findings should be interpreted in their specific context for each study, due to the variability in reporting different elastography techniques (shear-wave elastography and strain elastography) and stiffness values reported in various units (m/s and kPa) in different clinical contexts (pancreatoduodenectomy and distal pancreatectomy). Future research should emphasize the use of standardized elastography methods and uniformity in reporting results.

Two studies evaluated the role of intraoperative elastography in focal pancreatic lesion characterization and the impact on surgery guidance [37,38]. In the first study by Platz et al., 54 patients who underwent pancreatic surgery between 2017 and 2019 were retrospectively analyzed [37]. The study evaluated shear-wave speed and stiffness of pancreatic lesions; ultrasound values were further correlated with postoperative histopathological findings. According to the results, at cut-off values of 3 m/s and 28.7 kPa, sensitivity in detecting pancreatic cancer was 74%, with a specificity of 46.7%, a PPV of 78.4% and an NPV of 41.2% (*p* < 0.05) [37]. In this study, both SWE and CEUS were performed for the pancreatic regions of interest [37]. The authors concluded that intraoperative SWE is a valuable technique in PC detection and pancreatic lesions characterization, being able to identify small, previously undiagnosed lesions, aspects that finally lead to a more accurate pancreatic surgery [37].

The study performed by Elias et al. only included six patients. Surgery was performed in five out of six patients (with missing information regarding the patient for whom surgery was not performed). All neoplastic lesions were identified as “hard mass lesions” in strain elastography (SE), and, in one case, elastography was found to influence pancreatic surgery strategy, downstaging the pancreatic lesion. No further statistical analysis has been performed; the study was limited to confirming the existence of an increased stiffness of pancreatic tumors [38].

## 4. Discussion

Intraoperative ultrasound (IOUS) in PC surgery is gaining more significance due to its benefits: improved tumor localization, detection of previously undiagnosed PC lesions, and assessment of surgical margins [21,29]; in addition, IOUS was found to influence surgical strategy in a percentage reaching up to 49–61% of the cases [31,32]. However, few studies have analyzed the use of intraoperative elastography in pancreatic surgery.

Intraoperative elastography is a modern ultrasound technique that can provide valuable information during pancreatic surgery [37]. Intraoperative elastography provides quantifiable and reproducible information in the characterization of pancreatic lesions, an aspect that can improve PC staging, and can have an important contribution in the detection of small pancreatic lesions and surgical guidance [2,4,9]. In addition to standard greyscale ultrasound, elastography allows for real-time tumor characterization and assessment of tissue stiffness [21]. Elastography has been previously used intraoperatively for liver tumors [5], where the method was found to be able to properly differentiate benign from malignant lesions and characterize the degree of liver fibrosis [6,7]. In PC, EUS elastography is being used for malignant-benign distinction and for the evaluation of pancreatic fibrosis [2,10].

Known as a frequent complication after pancreatic surgery, POPF is more frequently encountered in the presence of soft pancreatic tissue [34,35]. Evaluation of pancreatic stiffness is currently performed via intraoperative palpation by the surgeon [34,35]. Pancreatic firmness is inversely correlated with the risk of POPF occurrence [35]. Intraoperative evaluation of the pancreas through the surgeon’s palpation is a subjective method, lacking objective elements, an aspect that limits the ability to reach valid scientific conclusions. Thus, intraoperative elastography of the pancreas could be a valuable tool to objectively assess pancreatic texture and its stiffness to better evaluate the risk of POPF.

This study analyzed existing data in the literature, and intraoperative elastography of the pancreas was used to predict POPF in pancreatic surgery.

In the study performed by Wadda et al., a SWE index was determined after intraoperative ultrasound elastography of the pancreas and was further analyzed to see if POPF could be predicted [1]. According to the results, patients with lower values of the SWE index (corresponding to a softer pancreatic tissue) have a higher risk of POPF, yet the results did not reach statistical significance (*p* > 0.005) [1]. The study was conducted on 15 patients who underwent pancreatoduodenectomy for pancreatic neoplasms. Three POPFs were documented (three Grade A and one Grade B). Intraoperative SWEI was inversely correlated with daily output of pancreatic juice (Spearman’s correlation test, *p* < 0.005) [1]. However, intraoperative SWE index positively correlated with preoperative SWE index measurements (*p* < 0.001) (both pre- and intraoperative elastography were performed) [1].

In order to better evaluate the use of intraoperative elastography in characterizing pancreatic texture, some authors expressed their results as Young’s module of pancreatic SWE; even if the value of Young’s module in intraoperative use of elastography was higher in the tumor group 16.0 kPa (11.4–19.2 kPa) than in control group 13.2 kPa (11.5–15.5 kPa), results were not statistically significant (*p* = 0.905, *p* > 0.05) [41].

More recently, Kawabata et al. performed a prospective study on a larger number of patients than the aforementioned authors [39]. According to their results, intraoperative pancreatic elastography can be used to predict POPF at a value of 2.2 m/s or less, and the histopathological results correspond to a softer pancreatic tissue [39]: AUC 0.797, sensitivity 85%, specificity 60.7%, PPV 60.7%, and NPV 85% [39].

Another study that supports the diagnosis of soft pancreatic texture by intraoperative ultrasonography is the one conducted by Hatano et al. [40]. After intraoperative analysis of 41 patients that undergo pancreatoduodenectomy (n = 30) and distal pancreatectomy (n = 11), a value of less than 2.09 m/s on elastography (value determined at the resection site), was found to significantly correlate with a higher risk of POPF occurrence in pancreatoduodenectomy group (and not in the distal pancreatectomy group, *p* = 0.09) with a sensitivity of 0.909, a specificity of 0.562 (*p* = 0.02) and area under the curve (AUC) of 0.739; however, the results did not reach statistical significance in multivariate analysis (*p* > 0.05) [40].

Both Hatano et al. and Kawabata et al. managed to confirm that intraoperative elastography values of less than 2.09 m/s and 2.2 m/s, respectively, are predictive risk factors for POPF after pancreatoduodenectomy interventions [39,40]. Results were not confirmed in distal pancreatectomies by Hatano et al. [40]. Yet, in the study performed by Hatano et al., results were not sustained after multivariate analysis [40].

Thus, the only study that managed to identify intraoperative elastography values of 2.2 m/s or less as an independent risk factor for POPF after pancreatoduodenectomy performed for PC was the one performed by Kawabata et al. [39].

Across all studies, pancreatic stiffness was measured using different elastography techniques; despite different units, all studies reported that a softer pancreatic tissue (lower strain ratio or lower velocity expressed as m/s) is associated with a higher risk of POPF.

Intraoperative elastography provides quantifiable and reproducible information for characterization of pancreatic lesions, an aspect that can improve PC diagnosis, and can have an important contribution in the detection and characterization of small pancreatic lesions [27,28]. The only study identified to be able to characterize intraoperative SWE values and significantly correlate them with histopathological benign or malignant results was the one performed by Batista da Silva et al. [30]. According to the results, detection of pancreatic cancer through intraoperative SWE is possible at cut-off values of 3 m/s and 28.7 kPa (*p* < 0.05).

Due to the early stages of research in the field, studies included in this manuscript have small sample sizes and are single-center studies, which represents a limitation of this paper. Future studies, with larger populations, are needed to confirm these results. In addition, studies that rely only on univariate analysis should be interpreted in the light of this limitation, and cut-off values should not be interpreted as independent predictors but as preliminary and intended to generate a hypothesis, rather than definitive; further studies that use standardized multivariate analysis to better assess the independent predictive value of these parameters are needed.

Direct comparison of the results between studies should not be attempted, and findings should be interpreted in their specific context for each study, due to the variability in reporting different elastography techniques (shear-wave elastography and strain elastography) and stiffness values reported in various units (m/s and kPa) in different clinical contexts (pancreatoduodenectomy and distal pancreatectomy). Future research should emphasize the use of standardized elastography methods and uniformity in reporting results.

In conclusion, cut-off values reported in the studies should be interpreted as exploratory and should represent a starting point for further studies aimed at validating their clinical implementation.

## 5. Conclusions

Intraoperative elastography can be a promising tool in the characterization of solid pancreatic lesions and can differentiate between benign and malignant tumors, but further studies on larger cohorts of patients are needed so that results with greater statistical power can be obtained. Detection of pancreatic cancer through intraoperative SWE is possible at cut-off values of 3 m/s and 28.7 kPa (*p* < 0.05). Values of 2.2 m/s or less obtained at intraoperative pancreatic elastography correspond to a soft texture of the pancreas and are considered an independent risk factor for POPF. Standardization of the above-mentioned ultrasound technique could be of real benefit, considering the rise in minimally invasive pancreatic surgery.

## Figures and Tables

**Table 1:** Risk of bias and applicability judgement (QASD-2) for included studies.

Study Risk of Bias/ Concerns of Applicability	Year	Patient Selection	Index Test	Reference Standard	Flow and Timing
Batista da Silva et al. [37]	2021	Low/Low	Unclear/Low	Low/Low	Low/NA
Elias et al. [38]	2011	Unclear/Low	High/Low	Unclear/Low	Low/NA
Kawabata et al. [39]	2020	Low/Low	Unclear/Low	Low/Low	Low/NA
Hatano et al. [40]	2015	Low/Low	High/Low	Low/Low	Low/NA
Yang et al. [41]	2023	Low/Low	Low/Low	Low/Low	Low/NA
Wada Y et al. [1]	2021	Low/Low	Unclear/Low	Low/Low	Low/NA

**Table 2:** Intraoperative elastography in predicting POPF after pancreatic surgery.

Study	Year	Design	No. of Patients	Key Outcome: POPF	Results/Cut-Off Values	Sensitivity/PPV (%)	Specificity/NPV (%)	*p*-Value
Kawabata et al., *Pancreas* (2020) [39]	2016–2018	P	48	+	2.2 m/s	85/60.7	60.7/85	*p* = 0.003
Hatano et al., *Int Surg* (2015) [40]	2010–2013	P	41	+	2.09 m/s			*p* = 0.02
Yang et al., *Chin J Hepatic Surg* (2023) [41]	2019–2020	R	19	+	16 kPa	-	-	*p* > 0.05
Wada et al., *Anticancer Res* (2021) [1]	2015–2019	R	15	+	16.06 kPa			*p* > 0.05

POPF = postoperative pancreatic fistula; PPV = positive predictive value, NPV = negative predictive value, R = retrospective, P = prospective.

**Table 3:** Intraoperative elastography in differentiating benign vs. malignant pancreatic lesions.

Study	Year	Design	No. of Patients	iSWE or iSE	Key Outcome: PC	Results/ Cut-Off values	Se/PPV (%)	Sp/NPV (%)	*p*-Value
Platz Batista da Silva et al., *J Ultrasound Med* (2021) [37]	2017–2019	R	54	iSWE	+	28.7 kPa/ 3 m/s	74/78.4	46.7/41.2	*p* < 0.05
Elias et al., *Pan American Health Care Exchanges* (2011) [38]	2010–2011	P	6	iSE	+	-	-	-	-

PC = pancreatic cancer; Se = sensitivity, Sp = specificity, PPV = positive predictive value, NPV = negative predictive value, R = retrospective, P = prospective, iSWE = intraoperative shear-wave elastography, iSE = intraoperative strain elastography.

## Data Availability

The original contributions presented in this study are included in the article. Further inquiries can be directed to the corresponding author.

## Abbreviations

The following abbreviations are used in this manuscript:

PC	Pancreatic cancer
POPF	Postoperative pancreatic fistula
SE	Strain elastography
SWE	Shear-wave elastography

## References

[1] Wada Y., Aoki T., Fujimori A., Ohike N., Koizumi T., Kusano T., Matsuda K., Nogaki K., Tashiro Y., Hakozaki T. (2021). Intraoperative Shear Wave Elastography as a Quantitative Predictor of Pancreatic Fibrosis and Exocrine Function. Anticancer Res..

[2] Hirooka Y., Kuwahara T., Irisawa A., Itokawa F., Uchida H., Sasahira N., Kawada N., Itoh Y., Shiina T. (2015). JSUM ultrasound elastography practice guidelines: Pancreas. J. Med. Ultrason..

[3] Society of Radiographers, British Medical Ultrasound Society (2022). BMUS Guidelines for Professional Ultrasound Practice. Seventh Edition. https://www.sor.org/getmedia/6d21e16f-95bb-4017-abb3-04757de4b255/SoR-and-BMUS-guidelines-2022-7th-Edv2-0?utm_source=chatgpt.com.

[4] Kawada N., Tanaka S. (2016). Elastography for the pancreas: Current status and future perspective. World J. Gastroenterol..

[5] Kato K., Sugimoto H., Kanazumi N., Nomoto S., Takeda S., Nako A. (2008). Intraoperative application of real-time tissue elastography for the diagnosis of liver tumours. Liver Int..

[6] Bartoș A., Iancu I., Ciobanu L., Badea R., Spârchez Z., Bartoș D.M. (2021). Intraoperative ultrasound in liver and pancreactic surgery. Med. Ultrason..

[7] Inoue Y., Takahashi M., Arita J., Aoki T., Hasegawa K., Beck Y., Makuuchi M., Kokudo N. (2010). Intra-operative freeahand real-time elastography for small focal liver lesions: Virtual palpation for non-palpable tumors. Surgery.

[8] Zaro R., Lupșor-Platon M., Cheviet A., Badea R. (2016). The pursuit of normal reference of values of pancreas stiffness by using Acoustic Radiation Force Impulse (ARFI) elastography. Med. Ultrason..

[9] Goertz R.S., Schulderer J., Strobel D., Pfeifer L., Neurath M., Wildner D. (2016). Acoustic radioation force impulse shear wabe elastography (ARFI) of acute and chronic pancreatitis and pancreatic tumor. Eur. J. Radiol..

[10] Iglesias-Garcia J., Larino-Noia J., Abdulkader I., Forteza J., Dominguez-Munoz J.E. (2010). Quantitative endoscopic ultrasound elastography: An accurate method for the differentiation of solid pancreatic masses. Gastroenterology.

[11] Pei Q., Zou X., Zhang X., Chen M., Guo Y., Luo H. (2012). Diagnostic value of EUS elastography in differentiation of benign and malignant solid pancreatic masses: A meta-analysis. Pancreatology.

[12] Mei M., Ni J., Liu D., Jin P., Sun L. (2013). EUS elastography for diagnosis of solid pancreatic masses: A meta-analysis. Gastrointest. Endosc..

[13] Belfiori G., De Stefano F., Tamburrino D., Gasparini G., Aleotii F., Camisa R.P., Arcangeli C., Lena M.S., Pecorelli N., Palumbo D. (2025). Anatomically resectable versus biologically borderline resectable pancreatic cancer definition: Refining the border beyond anatomical criteria and biological agressiveness. BJS Open.

[14] Siegel R.L., Miller K.D., Ahmedin Jemal D.V.M. (2020). Cancer statistics. CA Cancer J. Clin..

[15] Rahib L., Wehner M.R., Matrisian L.M., Nead K.T. (2021). Estimated projection of US cancer incidence and death to 2040. JAMA Netw. Open.

[16] Stoop T.F., Seelen L.W.F., van Land F., Lutchman K., van Dieren S., Lips D., van der Harst K., Kazemier G., Patijn G., de Hingh I. (2024). Nationwide use and outcome of surgery for locally advanced pancreatic cancer following induction chemotherapy. Ann. Surg. Oncol..

[17] Ren S., Song L.-N., Tian Y., Wang Z.-Q. (2025). Serum exosomal has-let-7f-5p: A potential diagnostic biomarker for metastatic pancreatic cancer detection. World J. Gastroenterol..

[18] Mizrahi J.D., Surana R., Valle J.W., Shroff R.T. (2020). Pancreatic cancer. Lancet.

[19] Soweid A.M. (2017). The bordeline resectable and locally advanced pancreatic ductal adenocarcinoma: Definition. Endosc. Ultrasound.

[20] Park W., Chawla A., Oreily E.M. (2021). Pancreatic cancer: A review. JAMA.

[21] Kotb A., Hafeji Z., Jesry F., Lintern N., Pathak S., Smith A.M., Lutchman K., de Bruin D., Hurks R., Heger M. (2024). Intra-Operative Tumour Detection and Staging in Pancreatic Cancer Surgery: An Integrative Review of Current Standards and Future Directions. Cancers.

[22] Strobel O., Neoptolemos J., Jager D., Buchler M.W. (2019). Optimizing the ouctomes of pancreatic cancer surgery. Nat. Rev. Clin. Oncol..

[23] Maggino L., Mallea G., Marchegiani G., Viviani E., Nessi C., Cipriani D., Esposito A., Landoni L., Casetti L., Tuveri M. (2019). Outcomes of primary chemotherapy for borderline resectable and locally advanced pancreatic ductal adenocarcinoma. JAMA Surg..

[24] Kizy S., Altman A.M., Wirth K.M., Marmor S., Hui J.Y.C., Tuttle T.M., Lou E., Amin K., Denbo J., Jensen E.H. (2020). Systemic therapy without radiation may be appropiate as neoadjuvant therapy for localized pancreas cancer. Hepatobiliary Surg. Nutr..

[25] Michiels N., Doppenberg D., Goren J.V., van Veldhuisen E., Bonsing B.A., Busch O.R., Stijn A., Crobach L.P., van Delden O.M., van Dieren S. (2023). Intraoperative Ultrasound During Surgical Exploration in Patients with Pancreatic Cancer and Vascular Involvement (ULTRAPANC): A Prospective Multicenter Study. Ann. Surg. Oncol..

[26] Zins M., Matos C., Cassinotto C. (2018). Pancreatic adenocarcinoma staging in the era of preoperative chemotherapy and radiation therapy. Radiology.

[27] Habib J.R., Kinny-Koster B., van Oosten F., Javed A.A., Cameron J.L., Lafaro K.J., Burkhart R.A., Burns W.R., He J., Thompson E.D. (2021). Periadventiceal dissection of the superior mesenteric artery for locally advanced pancreatic cancer: Surgical planning with the halo sign and string sign. Surgey.

[28] van Veldhuisen E., Walma M.S., van Rijssen L.B., Busch O.R., Bruijnen R.C.G., van Delden O.M., Mohammad N.H., de Hingh I.H., Yo L.S., van Laarhoven H.W. (2019). Added valuea of intra-operative ultrasound to determine the resectability of locally advanced pancreatic cancer following FOLFIRINOX chemotherapy (IMAGE): A propective multicenter study. HPB.

[29] Huscher C.G.S., Lazzarin G., Marchegiani G., Boggi U. (2024). Intraoperative intraductal ultrasonography of the main pancreatic duct during pancreatoduodenectomy: Technical description of a pilot series. Updates Surg..

[30] Ali E.S., Sharker S.M., Islam M.T., Khan I.N., Shaw S., Rahman A., Uddin S.J., Shill M.C., Rehman S., Das N. (2021). Targeting cancer cells with nanotherapeutics and nanodiagnostics: Current status and future perspectives. Semin. Cancer Biol..

[31] Lubner M.G., Gettle L.M., Kim D.H., Ziemlewicz T.J., Dahiya N., Pickhardt P. (2021). Diagnostic and procedural intraoperative ultrasound: Technique, tips and tricks for optimizing results. Br. J. Radiol..

[32] Mulder B.G.S., Feshtali S., Sarasqueta A.F., Vahrmeijer A.L., Swijnenburg R.-J., Bonsing B.A., Mieog J.S. (2019). A Prospective Clinical Trial to Determine the effect of Intraoperative Ultrasound on Surgical Strategy and Resection Ouctome in Patients with Pancreatic Cancer. Ultrasound Med. Biol..

[33] De Werra C., Quarto G., Aloia S., Perrotta S., Del Giudice R., Di Filippo G., Furino E., Amato B., Benassai G. (2015). The use of intraoperative ultrasound for diagnosis and stadiation in pancreatic head neoformations. Int. J. Surg..

[34] von Ehrlich-Treuenstaat V., Guenther M., Ilmer M., Knoblauch M.M., Koch D., Clevert D.A., Ormanns S., Klauschen F., Niess H., DHaese J. (2024). Preoperative ultrasound elastography for postoperative pancreatic fistula prediction after pancreatoduodenectomy: A prospective study. Surgery.

[35] Sushma N., Gupta P., Kumar H., Sharma V., Mandavdhare H., Kumar-M P., Nada R., Yadav T.D., Singh H. (2020). Role of ultrasound shear wave elastography in preoperative prediction of pancreatic fistula after pancreaticoduodenectomy. Pancreatology.

[36] Probst P., Huttner F., Meydan O., Hilal M.A., Adham M., Baretto S.G., Besselink M., Busch O.R., Bockhorn M., Del Chiaro M. (2021). Evidence Map of Pancreatic Surgery—A living systematic review with meta-analysis by the International Study Group of Pancreatic Surgery (ISGPS). Surgery.

[37] Platz Batista da Silva N., Engeser M., Hackl C., Brunner S., Hornung M., Schlitt H.J., Evert K., Stroszczynski C., Jung E.M. (2021). Intraoperative Characterization of Pancreatic Tumors Using Contrast-Enhanced Ultraspund and Shear Wave Elastography for Optimization of Surgical Strategies. J. Ultrasound Med..

[38] Elias J., Mauad F.M., Muglia V.F., Caetano E., dos Satos J.S., Kemp R., Pavan T., Carneiro A. Intraoperative application of real-time tissue elastography for the diagnosis and staging of pancreatic tumours. Proceedings of the 2011 Pan American Health Care Exchanges.

[39] Kawabata Y., Okada T., Iijima H., Yoshida M., Iwama H., Xu J., Hatano E., Fujimoto J., Suzumura K. (2020). Intraoperative Ultrasound Elastography Is Useful for Determining Pancreatic texture and Predicting Pancreatic Fistula After Pancreaticoduodenectomy. Pancreas.

[40] Hatano M., Watanabe J., Kushihata F., Tohyama T., Kuroda T., Koizumi M., Kumagi T., Hisano Y., Sugita A., Takada Y. (2015). Quantification of pancreatic stiffness on intraoperative ultrasound elastography and evalutaion of its relationship with postoperative pancreatic fistula. Int. Surg..

[41] Yang T., Han W., Qui F., Qi J. (2023). Evaluation of pancreatic texture by intraoperative ultrasonic elastography. Chin. J. Hepatic Surg. (Electron. Ed.).

